# Supplementary figures and images for: Kaposi’s sarcoma-associated herpesvirus promotes mesenchymal-to-endothelial transition by resolving the bivalent chromatin of PROX1 gene
Source: PLoS Pathog. 2021 Sep 7;17(9):e1009847. doi: 10.1371/journal.ppat.1009847 (PMC8448337; doi:10.1371/journal.ppat.1009847)

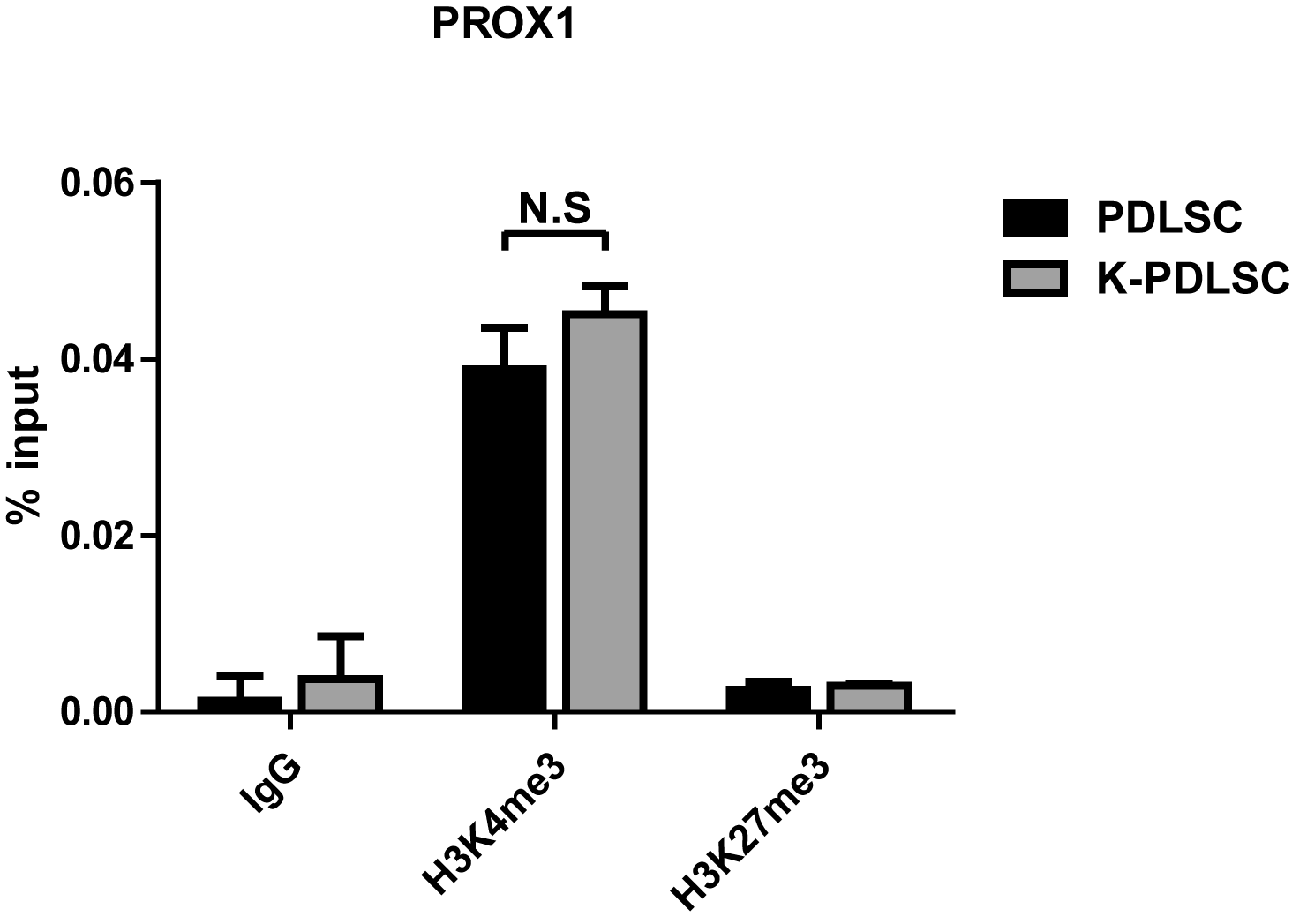

Supplement: S1 Fig — Mock- and KSHV-infected PDLSCs were subjected to a Chromatin immunoprecipitation (ChIP) assay with antibodies against H3K4me3 and H3K27me3. The extracted DNA was analyzed by qPCR with primers of the PROX1 coding region. (TIF) [file ppat.1009847.s001.tif]

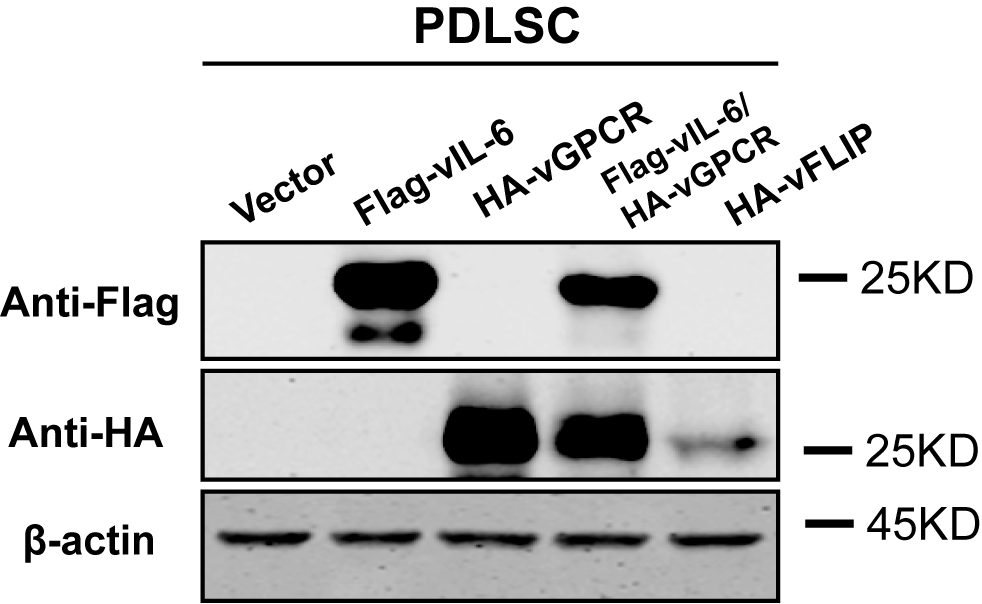

Supplement: S2 Fig — PDLSCs were transfected with a class of viral gene expression vectors, including K1, K12, ORF45, ORF50, ORF71, ORF73, K8, PAN, vIL-6, and vGPCR (Fig 5). Among them, vIL-6, vGPCR and vFLIP were tagged with HA or Flag tags. The expression of vIL-6, vGPCR and vFLIP were examined by Western blot. (TIF) [file ppat.1009847.s002.tif]

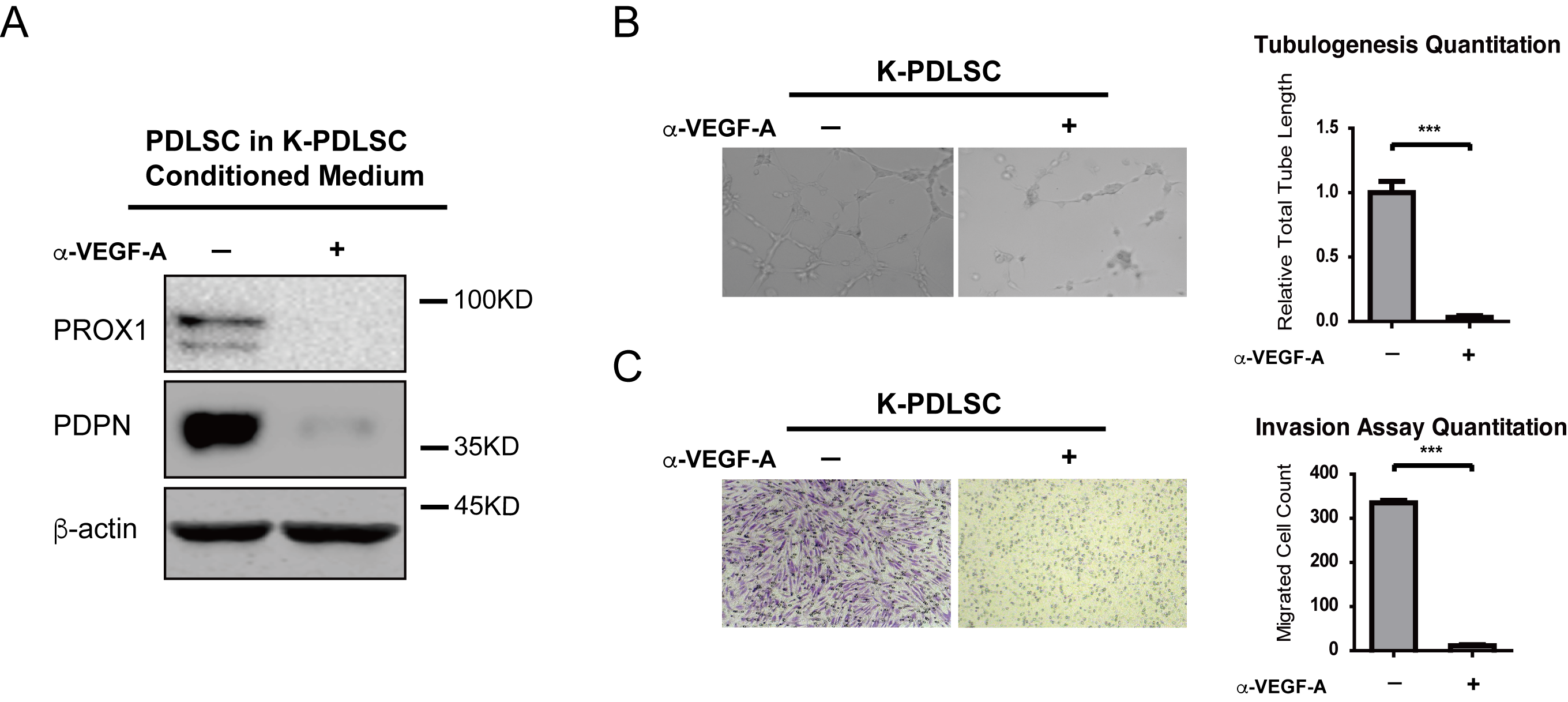

Supplement: S3 Fig — (A) PDLSCs were treated with conditioned medium from KSHV-PDLSC. The expression of PROX1 and PDPN were analyzed by Western blot. (B) KSHV-infected PDLSCs (K-PDLSCs) were placed on Matrigel, and tubulogenesis was examined by Tubule formation assay and quantified by measuring the total segment tube length using the ImageJ software. (C) K-PDLSCs (1.5x10^4^ cells/well) were seeded in the upper chamber of Transwell with Matrigel in serum-free α-MEM. α-MEM containing 20% FBS was used as a stimulus for chemotaxis in the lower chamber. Cells migrated to the lower chamber were stained with crystal violet. The error bars represent SD. Statistics analysis was performed using t-test in GraphPad Prism. P-values < 0.05 were considered significant (*P<0.05, **P<0.01 and ***P<0.001) and N.S represented no significance. (TIF) [file ppat.1009847.s003.tif]
