# Supplementary material for: Kaposi’s sarcoma-associated herpesvirus promotes mesenchymal-to-endothelial transition by resolving the bivalent chromatin of PROX1 gene
Source: PLoS Pathog. 2021 Sep 7;17(9):e1009847. doi: 10.1371/journal.ppat.1009847 (PMC8448337; doi:10.1371/journal.ppat.1009847)
Supplement: Attachment — Submitted filename: ResponsestoReview.docx [file ppat.1009847.s004.docx]

**Responses to the review on PPATHOGENS-D-21-00988**

Reviewer #1：

*This study described very interesting observation, which KSHV infection induces the mesenchymal-to-endothelial transition (MEndT) through induction of PROX1. PROX1 has recently been described as an important cellular factor for maintaining lytic phenotype of KSHV infection (Gramolelli S. et al., Cancer Res 2020; Choi D. et al., 202 Cancer Res 2020), and this study further adds to the previous studies that KSHV encoded proteins could induce PROX1 genes via increasing H3K4me3 and decreasing H3K27me3 marks on PROX1 promoter; this mechanism may establish suitable environment for maintaining KSHV life cycle. The authors also proposed interesting epigenetic model of “two-factor-authentication” for efficient and accurate response for activation of the putative pioneer factor. VIL-6 and vGPCR were found to be responsible for PROX1 up-regulation through paracrine fashion. Although number of samples was limited, the authors also included patients samples for the IHC staining to further demonstrate upregulation of PROX1 at KS lesions. This reviewer think that this study is conceptually novel, experiments are well-designed and controlled, conclusion is supported by the results.*

*1. Depending on resolution of ChIP-assay (fragmentations by sonication), the authors theory for “two-factor-authentication” may not be accurate. Prox1 promoter and a lncRNA, Prox1-AS, promoter localize very next each other (maybe partially overlapped), and very strong H3K27me3 peaks are often adjacent to strong H3K4me3 peaks in public ChIP-seq data sets (including both PEL and iSLK cells). There is a possibility that one promoter is regulated by vIL-6 and the other is regulated by GPCR pathway (not same nucleosome), and ChIP-assays with fixed DNA/histone complex might be precipitating adjacent nucleosomes. Of course, there is a possibility that PDLSCs indeed possess the bivalent histone mark and regulates the PROX1 promoter as the author suggested. For this reviewer, it is a little difficult to understand that activation of a nucleosome, which usually increases H3K27 acetylation via coactivator complex recruitment, did not reduces H3K27me3. Examining Prox1-AS expression in presence of vIL-6 and/or vGPCR, or designing additional primer sets that probe Prox1-AS promoter would clarify the result, if the authors like to do so. In minimum, the authors may expand the discussion to avoid a possible misleading.*

**Response:** Thank the reviewer for insightful suggestion. The bivalent domain in the PROX1 promoter was confirmed by a sequential ChIP assay, which is the gold standard assay for studying bivalent chromatin in embryonic cells, developed by Eric Lander’s lab and used by others. We agree with the reviewer in that the technique has some limitation especially about the resolution of ChIP assay. To clarify if there is any possibility that KSHV-mediated “dual signaling” actually regulates two different promoters (PROX1 and adjacent noncoding RNA Prox1-AS), we analyze the effect of KSHV on Prox1-AS RNA transcription. A pair of primers probed Prox1-AS promoter was designed to examine the Prox1-AS expression in mock- and KSHV-infected PDLSCs. We found that Prox1-AS expression is not response to KSHV infection, therefore ruling out the possibility KSHV-mediated two signalings act on two different genes (see the figure below).

*2. For ChIP-assays, it would be important to include other genomic regions as controls in order to demonstrate relative enrichment of* *H3K4me3 and H3K27me3 marks on the PROX1 promoter.*

**Response:** We performed a ChIP assay with the probes targeted the coding sequence (CDS) of PROX1 and found that there was only the enrichment of H3K4me3 marks but no H3K27me3 on the PROX1 coding sequence region, which did not altered in response to KSHV infection. This result is included in the manuscript (Supporting Information, Fig. S1). In addition, other genomic loci, including PML, TGFBR2, and TGFB3, were also included in ChIP assay and found no bivalent domain.

*3. Fig. 3C, do the authors expect to see stronger H3K4me3 signals in KSHV infected cells (K27/K4)? Please clarify. Line 721 for the legend, it should read qPCR (not RT-qPCR).*

**Response:** As the sequential ChIP was performed by continuous two-step ChIP assay, the yield of eluted DNA was much less than regular ChIP leading to the signals of histone marks in Fig. 3C relatively weaker compared to Fig. 3A.

In accordance to reviewer comment, “ RT-qPCR” has been changed to “qPCR” in revised manuscript (Page 36, line 786).

Reviewer #2：

*In this manuscript, Yao et al. revealed that KSHV infection of MSCs induced the expression of PROX1, which plays a critical role in the process of Mesenchymal-to-Endothelial transition. Mechanistically, the authors found that KSHV infection promoted the expression of PROX1 by resolving the bivalent chromatin; vIL-6 increased the active histone marker H3K4me3 by recruiting MLL2 and Set1 complex, and vGPCR-VEGF-A reduced the repressive marker H3K27me3 by removing EZH2. Overall, the study provides a novel model for the epigenetic regulation of Prox1 by KSHV and most data are convincing and well presented.*

*1. All WB crops are lack of molecular weight labeling.*

**Response:** Thank the reviewer for the constructive suggestion. We have labelled molecular weight markers in Western blots.

*2. Some statistical data are lack of significance evaluation (e.g., Fig 2C and D, list is not exhaustive.)*

**Response:** We completed the statistical evaluation for all data in revised manuscript.

*3. Fig5A, it would be more convincing if authors could show the expression data of the indicated KSHV genes. ORF71 seemed to slightly induce PROX1 and greatly promoted the expression of PDPN, while vIL-6 and vGPCR affected Prox1 more specifically. Authors may provide some explanation.*

**Response:** Thank the reviewer for the insightful advice and consideration. Given that there was short of viral protein antibodies, we were not able to check the expression of each viral protein. However, in the constructs of vIL-6, vGPCR, and vFLIP, these viral proteins were labeled with an HA or Flag tag so that we are able to examine the expression of these three proteins with Western using tag antibodies. The expression of vIL-6, vGPCR and vFLIP in the experiment is included in Supporting Information, Fig. S2.

Our laboratory has explored ORF71 for its role in KSHV-mediated MEndT and found that the regulation of PDPN by ORF71 mainly through NF-kB signaling pathway other than epigenetic regulation of Prox1. A manuscript entitled “Kaposi’s sarcoma-associated herpesvirus vFLIP promotes MEndT to generate hybrid M/E state for tumorigenesis” has been submitted to PLoS Pathogens and is currently under revision.

*4. Figure 7G and H showed the effect of ectopically expressed vIL-6 and vGPCR, and it would be better to present these data in Figure 6. On the other hand, here authors would better show how ablation of vIL-6 and/or vGPCR in K-PDLSC affects tube formation and invasion.*

**Response:** Thanks for the constructive suggestions. In compliance with the first suggestion, the figure 7G and H have been moved to Figure 6 (Fig. 6H and I in revised manuscript). In compliance with the second suggestion, we employed a neutralizing antibody against VEGF-A to ablate vGPCR-VEGF-A axis and found that the tube formation and invasion were dramatically reduced in K-PDLSC, vGPCR and vGPCR/vIL-6 expressing PDLSCs. The results are included in revised manuscript (Figure 7G and H, Supporting Information Fig. S3). As there is no antibody against vIL-6 commercially available, we were only able to perform the experiment with VEGF-A neutralizing antibody.

*5. Fig7C, vGPCR is a lytic gene and this reviewer noted that the knockdown was relatively inefficient. Authors may consider including PDLSC control to show that vGPCR is indeed expressed in K-PDLSC.*

**Response:** Thank the reviewer for the careful consideration. We made statistical analysis that showed the knockdown efficiency for vGPCR was extremely notable compared to control (si-NC) (Fig. 7C). In addition, the RT-PCR parameters indicate that vGPCR gene is expressed in K-PDLSCs, so is vIL-6 (see below).

*6. Although authors showed that siRNA-mediated knockdown of vIL-6 and vGPCR abolished the expression of Prox1 in K-PDLSC, it would be more convincing if authors could use knockout virus to confirm the observation.*

**Response:** Thanks for the insightful advice. Given the current data, it has been no doubt that the efficiency of siRNA-mediated knockdown of vIL-6 and vGPCR was sufficient to abolish the regulation of KSHV infection on Prox1 in K-PDLSC, suggesting that there was a simpler way to achieve the goal as construction of a knockout virus is a magnificent project.

Reviewer #3：

*Understanding the molecular mechanisms of Kaposi’s sarcomagenesis in the right cellular context is still an important gap in our knowledge of KSHV oncobiology. The present manuscript represents a significant and novel contribution to this field. … … . This paper is a significant contribution to our understanding of the molecular mechanisms of KSHV oncogenesis in the context of mesenchymal stem cell progenitors. It reinforces the critical and necessary role of KSHV angiogenic oncogenes such as vIL-6 and vGPCR in the early stages of KSHV sarcomagenesis.*

*1. In Figure 6 it will be interesting to show as much as possible the experimental systems of Figure 1 to show the extent of the contributions of vIL-6 and vGPCR to the endothelial-lineage and LEC phenotypes shown in Figure 1, particularly the western blots showing the endothelial markers.*

**Response:** Thank the reviewer for the insightful suggestion. The expression changes of endothelial-lineage and LEC specific markers in PDLSCs in response to vIL-6 and vGPCR has been examined using Western blot and the result has been added in revised manuscript (Fig. 6G).

*2. The regulation of PROX1 by vGPCR via VEGF is interesting, particularly in published evidence showing that vGPCR signaling and oncogenicity become potentiated in the LEC lineage. It will be interesting to determine whether there is a mutual regulation of vGPCR by PROX1 in this system as well.*

**Response:** This is a good question. Berenice et al. has reported that PROX1 could be upregulated by KSHV, which is crucial to activate vGPCR-enhanced cell proliferation, migration, VEGF expression, and tumor formation. Recently, two articles by Gramolelli et al. and Choi et al reported that KSHV-promoted PROX1 activation enhances KSHV lytic replication and sustains the population of KSHV infected cells that would otherwise be lost as KS cells divide. In this study, we found that vGPCR induced the expression of PROX1 through epigenetic regulation of the PROX1 promoter. Therefore, evidence has suggested a mutual regulation of vGPCR by PROX1. A discussion on this issue has been added to the Discussion section of revised manuscript (page 17, lines 362-364).

*3. The autocrine mechanisms proposed in Figure 6 are intriguing. These data would be reinforced by using blocking antibodies to the VEGF and IL-6 receptors.*

**Response:** Thanks for the constructive suggestion. We employed a neutralizing antibody against VEGF-A to block vGPCR-VEGF-A axis and found that PROX1 expression was blocked and the tube formation and invasion were dramatically reduced in K-PDLSC, vGPCR and vGPCR/vIL-6 expressing PDLSCs. The results are included in revised manuscript (Supporting Information, Fig. S3; Figure 7G and H).

**Minor Issues: Editorial and Data Presentation Modifications**

Reviewer #1：

*1. Please double check accuracy of references. For example, Reference 20 (Page 6) does not match what the paper describes.*

**Response:** Thanks for pointing out the error. We have checked the accuracy of references and corrected the careless mistake.

*2. It is important to include two recent studies regarding function of PROX1 on KSHV lytic replication (above mentioned). These studies have direct association with the authors studies and add an exciting discussion point, which KSHV establishes suitable microenvironment for KSHV replication through PROX1 activation via vIL-6 and vGPCR.*

**Response:** These two recent publications regarding the function of PROX1 in KSHV lytic replication have been included in revised manuscript and the relevance to our current research is discussed in the Discussion section (page 17, lines 362-364).

Reviewer #3：

*1. The quality of some western blots that reflects the impact of silencing in key phenotypes may be improved, and its quantitative information could be reinforced by densitometric quantification of duplicates or triplicates.*

**Response:** The reviewer’s suggestion has been well taken. We have tried our best to improve the quality of the Western images and added quantitation numbers in the figures.
